# Anatomical Reconstruction of Severe Ectropion With Lazy Pentagonal Wedge Resection: A Case Report

**DOI:** 10.7759/cureus.101870

**Published:** 2026-01-19

**Authors:** Rania Vasiliki Kasimi, Dimitra Kyrana, Alexandros Kasimis, Ameer Shehade, Dimosthenis Chrysikos, Dimitrios Filippou, Theodore Troupis

**Affiliations:** 1 Department of Anatomy, Athens Medical School, National and Kapodistrian University of Athens, Athens, GRC

**Keywords:** aesthetic, anatomical reconstruction, ectropion repair, involutional ectropion, lazy pentagonal wedge resection, lower eyelid, plastic surgery

## Abstract

Involutional ectropion is defined as the acquired eversion of the eyelid margin, most frequently affecting the lower eyelid, that occurs due to age-related changes in the eyelid supporting structures. We present a novel application of the lazy pentagonal wedge resection technique as a stand-alone procedure for the correction of severe involutional ectropion. While the technique is traditionally described for excising eyelid margin tumors or lesions, its use as a primary method for involutional ectropion repair has not been substantially documented in the current literature. This case, therefore, explores a repurposed application of Lazy Pentagonal Wedge Resection. An 88-year-old male presented with a two-year history of severe unilateral right lower eyelid ectropion, classified as Grade 5L according to the Moe and Linder grading scale. The condition was associated with generalized facial laxity and chronic compensatory eyebrow elevation. Clinical examination revealed conjunctival injection due to chronic conjunctivitis, tarsal laxity, and compromised tissue integrity. Based on the clinical presentation, systemic comorbidities, patient history, and the patient’s monocular status, the lazy pentagonal wedge resection technique was selected as a stand-alone, technically straightforward approach, as it provides long-lasting and durable results, tarsal alignment, structural support, and lower eyelid tightening, while minimizing scarring, dog-ear deformity, tissue overworking, and the risk of reoperation. The procedure fully corrected the ectropion, resulting in complete functional and anatomical restoration and full resolution of symptoms, with no complications. Cosmetic outcomes were favorable, with no visible scarring or dog-ear deformity.

## Introduction

Ectropion is a pathological malposition of the eyelid characterized by eversion of the eyelid margin away from the globe. Ectropion most commonly affects the lower eyelid; however, upper eyelid involvement may occur in conditions associated with significant eyelid laxity, such as floppy eyelid syndrome [1]. Moreover, ectropion is traditionally categorized into cicatricial, congenital, paralytic, mechanical, and involutional forms, each with distinct etiologic factors and clinical implications [1,2].

Involutional ectropion arises primarily from age-related horizontal laxity of the lower eyelid [1]. Its prevalence among elderly individuals has been reported to reach approximately 2% [3]. Insufficient eyelid-globe apposition leads to several symptoms, including epiphora, photophobia, ocular irritation, foreign body sensation, and lower eyelid erythema [1,3]. Furthermore, prolonged malposition may result in exposure keratopathy, corneal scarring, and, ultimately, irreversible visual impairment. Correction of ectropion frequently requires surgical intervention to restore normal eyelid position, function, ensure structural support for compromised periocular tissues, and relieve symptomatic discomfort, while preventing long-term complications [1-3]. Appropriate surgical planning is essential and relies on accurately identifying the underlying etiology and the degree of horizontal and vertical eyelid laxity, in addition to completing a thorough clinical evaluation [1]. The cornerstone of involutional ectropion repair is horizontal eyelid margin tightening. [3,4]. Although several surgical techniques are available, including canthopexy, lateral or medial canthoplasty [1,5-7], and full-thickness lid margin resection, such as Bick’s procedure [7,8], their success depends on adequate tissue quality and structural integrity of the lower eyelid tissues [9]. Therefore, when these elements are compromised, recurrence of the ectropion and/or suboptimal functional and aesthetic outcomes can occur.

A modified curvilinear, or “lazy,” pentagonal wedge resection (LPWR) has been described as an alternative to the standard full-thickness pentagonal wedge resection. This technique maintains the ability to achieve precise tarsal alignment while reducing the risk of postoperative scarring and dog-ear deformity. The use of a curvilinear incision instead of a linear one may minimize cutaneous redundancy and improve cosmetic appearance by aligning the incision with relaxed skin tension lines [10].

Although the LPWR technique has been primarily applied for excision of lower eyelid neoplasms and lesions [10], to date and to our knowledge, it has not been previously documented as a primary repair for involutional ectropion. The present case report introduces a novel, repurposed application of this technique as a durable alternative for severe ectropion repair in selected high-risk patients with compromised tissues and severe eyelid laxity, expanding its role beyond lesion excision.

## Case presentation

An 88-year-old male presented with a two-year history of right lower eyelid ectropion. He reported persistent symptoms, including epiphora, ocular irritation, foreign body sensation, photophobia, and recurrent conjunctival inflammation. The patient was monocular, with vision preserved only in his right eye.

Additionally, the patient was noted to have a visually significant cataract in the right eye. He denied any history of facial nerve palsy, trauma, or previous eyelid surgery on his right eye. His medical history was notable for ongoing antiplatelet therapy.

**Figure 1:**
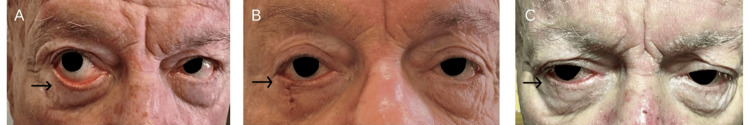
(A) Pre-operative view (B) One-week postoperative view (C) One-month postoperative view. (A) Pre-operative view: demonstrating severe lower eyelid ectropion with lateral predominance and moderate medial involvement; the arrow denotes the affected eye. (B) One-week postoperative view: showing eyelid tightening and reconstruction of the eyelid margin; the arrow denotes the affected eye. (C) One-month postoperative view: demonstrating complete reconstruction of the lower eyelid ectropion, scar maturation, and markedly reduced conjunctival edema secondary to chronic conjunctivitis; the arrow denotes the affected eye. Original photo by the authors. For General Data Protection Regulation compliance, the iris has been obscured bilaterally.

Upon examination, the patient exhibited severe ectropion affecting the lateral half of the lower eyelid and moderate ectropion involving the medial half (Figure 1A). The condition was classified as Grade 5L ectropion, in accordance with the Moe and Linder grading scale [11], indicating advanced severity with predominantly lateral involvement.

The ectropion was unilateral and associated with generalized facial laxity, including brow ptosis, which the patient reported had led to chronic compensatory elevation of the eyebrows. The patient also presented with conjunctival injection secondary to chronic conjunctivitis.

Eyelid laxity assessment revealed a positive distraction test with displacement equal to 9 mm, indicating significant canthal tendon laxity [12]. The snap-back test was also abnormal, as the lower eyelid failed to return to its normal position against the globe, leaving the ocular surface exposed and unprotected [12].

The inferior punctum was found to be malpositioned caudally and laterally. A Schirmer test score of 8 mm confirmed moderate ocular dryness, correlating with the patient’s complaints of foreign body sensation [13].

Examination demonstrated significant tarsal plate laxity and compromised lower eyelid tissue support. In light of these findings, the marked horizontal laxity and compromised structural integrity of the lower eyelid, surgical repair was deemed necessary (Figure 1A).

Surgical technique: lazy pentagonal wedge resection of eyelid margin

A lazy pentagonal wedge resection (LPWR) of the right lower eyelid margin was performed. LPWR enables the excision of redundant skin, orbicularis oculi muscle, tarsal plate, and gray line in a manner similar to full-thickness pentagonal wedge resections, while also facilitating proper tarsal alignment and lower eyelid tightening and minimizing postoperative scarring and the risk of cicatricial ectropion or dog-ear deformity [10].

Determination of Resection Dimensions

Using anatomical forceps, the lower eyelid was gently elevated upward towards the lateral canthus until it reached the approximate physiological position. The amount of excess tissue observed was used to determine the incision dimensions.

**Figure 2:**
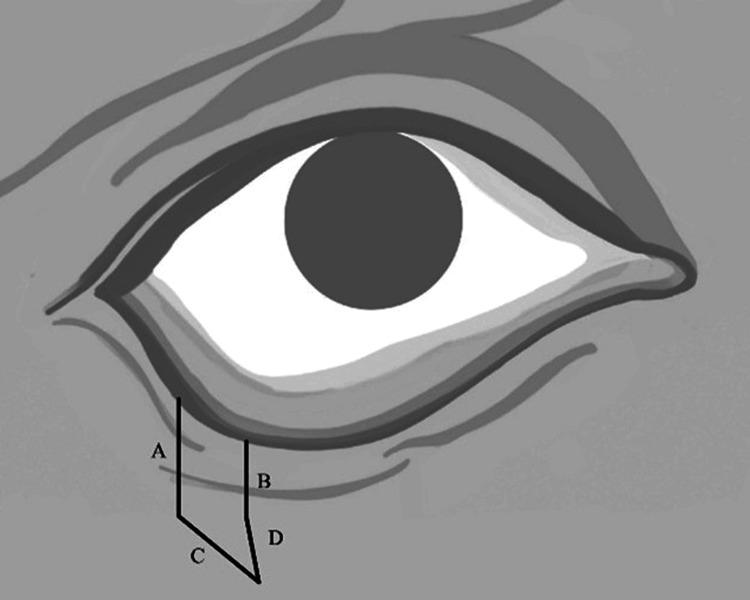
Schematic illustration of the lazy pentagonal wedge resection design. The incision includes two parallel vertical limbs (A, B) and an inferomedially oriented triangular component following the relaxed skin tension lines (C, D). Original schematic illustration by the authors.

**Figure 3:**
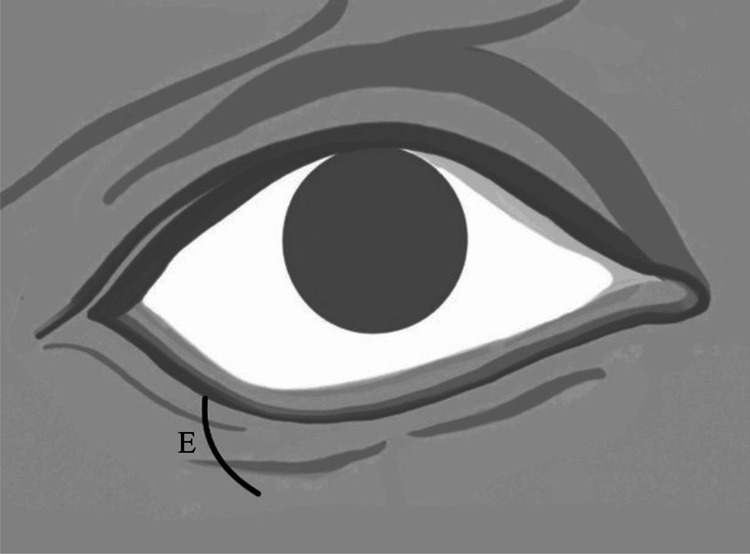
Schematic illustration showing the final curvilinear configuration (E) of the surgical wound and lower eyelid tissue reconstruction. After excision of the orbicularis oculi muscle, skin, tarsal plate and skin closure using the lazy pentagonal wedge resection technique, (E) represents the final curvilinear configuration of the surgical wound and lower eyelid tissue remodeling. Original schematic illustration by the authors.

The length of the vertical incisions was determined in accordance with the orbicularis oculi muscle laxity defined by the snap-back test. The triangular incision created to prevent dog-ear deformity had to remain within the lower eyelid, without exceeding the borders of the orbicularis oculi muscle. Therefore, a medially directed triangular incision instead of a vertical incision within the lower eyelid was selected to remain within the borders of the orbicularis oculi muscle and to align with relaxed skin tension lines (Figure 2, Figure 3).

Surgical Technique

**Figure 4:**
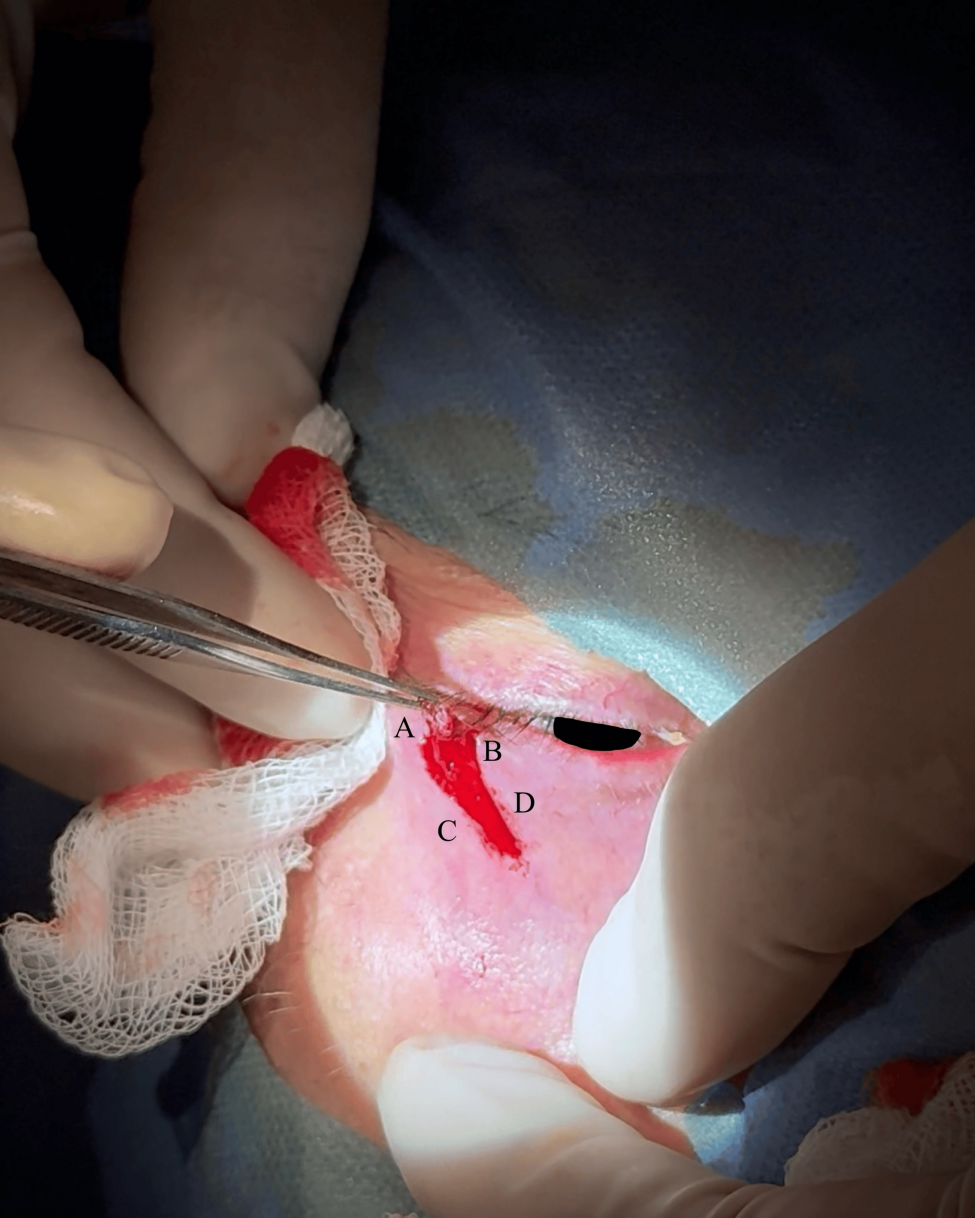
Intraoperative image showing the curvilinear lazy pentagonal wedge incisions of the lower eyelid margin, designed along relaxed skin tension lines before tarsal plate incision. The incision includes two parallel vertical limbs (A, B) and an inferomedially oriented triangular component following the relaxed skin tension lines (C, D) corresponding to the design illustrated in Figure 2. Original intraoperative photo by the authors. For General Data Protection Regulation compliance, the iris has been obscured.

The procedure was performed under local anesthesia using lidocaine with 2% adrenaline. A No. 15 blade produced two parallel vertical incisions at the lower eyelid margin: the lateral incision 4 mm from the lateral canthus, and the medial incision 4 mm from the lateral incision; both were extended inferiorly for 4 mm. Two additional incisions formed a triangular excision medially at approximately a 45-degree angle, with the inferior incisions angled inferomedially to converge. The medial incision was slightly longer than the lateral, and the incisions were curvilinear to align best with facial skin tension lines (Figure 4).

Skin and orbicularis oculi muscle were excised using straight iris scissors. Hemostasis was achieved via bipolar electrocautery. The lash line was aligned at the converging point inferomedially via skin closure using simple, interrupted 5-0 nylon sutures. The gray line endpoints of the initial vertical incisions were resected using straight iris scissors. The gray line was aligned using one simple, interrupted 5-0 nylon suture; the suture tails were left long to allow slight vertical traction on the eyelid margin. The tarsus was approximated with a buried 5-0 poliglecaprone 25 suture. The remaining lash line was aligned via skin closure using simple, interrupted 5-0 nylon sutures. All suture tails were left long and were secured in a caudal fashion beneath thin sterile adhesive tape to avoid ocular surface contact. Antibiotic corticosteroid ointment was applied to the conjunctiva to provide antimicrobial protection, moisture, and reduce preexisting edema due to chronic conjunctivitis. The eye was dressed with sterile gauze for 24 hours. Sutures were removed on postoperative day 6.

Follow-up

The operation achieved complete reconstruction of the lower eyelid with no complications. One-week postoperative findings (Figure 1B) showed satisfactory postoperative recovery, with improvement of lower eyelid position, reduced symptoms, and conjunctival injection.

One-month postoperative (Figure 1C) findings show an excellent surgical result with full correction of the ectropion of the lower eyelid and excellent wound healing. The procedure corrected the lower eyelid margin architecture.

The patient reported complete resolution of epiphora, ocular irritation, and foreign body sensation. The signs of chronic conjunctivitis were minimized. Finally, the cosmetic outcomes were favorable with no unsightly scarring or dog-ear deformity. 

No complications were present.

## Discussion

Involutional ectropion is an age-related eyelid malposition that can lead to chronic ocular surface exposure, chronic conjunctivitis, and, in advanced cases, vision-threatening exposure keratopathy [1,3]. The patient in this case presented with long-standing, severe unilateral ectropion in the context of marked horizontal laxity, generalized facial laxity, and monocular status. The compensatory mechanisms the patient used in response to the involutional ectropion, such as the additional habitual elevation of the eyebrows and frequent eye rubbing, contributed to the progressive worsening of the pre-existing ectropion over time. These factors, combined with conjunctival injection secondary to chronic conjunctivitis and reduced tear production, placed him at increased risk for progressive exposure keratopathy and loss of vision. Given the chronicity of symptoms, failure of conservative management, negative impact on his visual function, his overall quality of life, and high-risk profile, surgical intervention was deemed necessary to restore eyelid-globe apposition and to protect the only seeing eye [1].

It is worth mentioning that in all cases of tissue reconstruction in plastic surgery, a comprehensive understanding of regional anatomy is essential for the appropriate selection and precise application of surgical techniques. The lower eyelid anatomy is categorized into three principal lamellae: the anterior (skin and orbicularis oculi muscle), middle (orbital septum and lower lid fat pads), and posterior (tarsal plate, lower eyelid retractors, and conjunctiva) [14]. The tarsal plate - or tarsus - is of crucial importance in this case, as it houses the meibomian glands and provides physical form and structural support to the lower eyelid due to its dense, fibrocartilaginous anatomical structure. Tarsal repair, therefore, is vital regarding the functional reconstruction of the eyelid, and its compromised quality must be taken into consideration [15,16].

In the present case, LPWR was selected as a stand-alone, durable, safe, and straightforward procedure for the correction of severe involutional ectropion, allowing effective lid tightening with favorable functional and cosmetic outcomes in an anatomically compromised eyelid.

Several alternative techniques, such as the canthopexy and canthoplasty procedures, were considered but were deemed less suitable given the severity of tissue laxity, clinical presentation, comorbidities, and the patient’s monocular status. Furthermore, canthopexy reinforces and tightens the lateral canthal tendon without canthotomy or extensive tissue dissection, but it is suited for less-severe eyelid laxity [5,6]. Therefore, it is inadequate for advanced ectropion with pronounced tarsal and skin laxity, as in this case [5,6]. Lateral canthoplasty procedures, particularly the lateral tarsal strip (LTS), are widely used to address horizontal eyelid laxity and can effectively restore eyelid and punctal position [5]. However, LTS and related lateral canthal tightening procedures may alter the lateral canthal angle and reduce the peripheral visual field, a critical consideration in monocular patients [7]. In this specific case, the pronounced horizontal laxity and weakened tarsal plate render the LTS technique suboptimal. ‘Tightening’ the lid margin by only a partial resection or canthopexy remains a static repair that stabilizes the eyelid initially, but it fails to deliver enduring and lasting results, as the lower eyelid may eventually stretch again over time [4,9]

Additionally, lateral canthoplasty has been associated with higher reoperation rates and less favorable functional outcomes when compared with full-thickness lid margin resection, such as Bick’s procedure [8]. Bick’s procedure, a full-thickness lateral lid margin resection, shares the functional goals of LTS while being technically straightforward and effective for moderate horizontal laxity [8,17]. It has been shown to provide superior anatomical and functional results relative to LTS in some cohorts and to reduce the incidence of granuloma formation, which may be related to buried meibomian gland tissue in LTS constructs [8]. However, Bick’s procedure has recognized drawbacks, including potential rounding and medial displacement of the lateral canthal angle, as well as the risk of compromising peripheral vision-important limitations in monocular patients [7]. When large wedges are required for severe ectropion, Bick’s resection may also lead to dog-ear deformity and noticeable scarring, potentially affecting both function and cosmesis.

Furthermore, the LPWR technique was preferred over a full-thickness pentagonal wedge resection. A full-thickness pentagonal wedge resection is an established technique for treating a variety of eyelid disorders, including neoplasms, trichiasis, floppy eyelid syndrome, and horizontal laxity [10,17,18]. The standard design preserves tarsal continuity and minimizes lid margin notching; however, the vertical alignment of the incision often crosses relaxed skin tension lines, predisposing to conspicuous scarring [10]. Additionally, the inferior triangular component of the defect may create cutaneous redundancy and dog-ear deformity, sometimes necessitating secondary surgical revision [10]. These limitations become more clinically relevant in elderly patients for whom avoiding reoperation is critical, patients with severe horizontal laxity and/or compromised tissue integrity requiring larger resections with durable, long-term outcomes, and in patients for whom cosmetic results are of significant importance. The LPWR technique overcomes the aforementioned issues. Moreover, by employing a curvilinear configuration aligned with relaxed skin tension lines, the lazy design maintains the structural advantages of the traditional pentagonal wedge - namely robust tarsal shortening and precise lid margin realignment - while also reducing the risk of visible scarring and dog-ear formation [10].

Existing reports describe the use of LPWR predominantly for the excision of lower eyelid lesions and margin lesions rather than for primary correction of ectropion [10]. To our knowledge, this is the first report describing the LPWR as a stand-alone primary repair for severe involutional ectropion. Its novel application in this case demonstrates that the LPWR can serve as an effective and cosmetically favorable alternative to traditional ectropion procedures in patients with marked horizontal laxity and compromised tissue quality.

In this context, the lazy pentagonal wedge resection provided several advantages. The technique avoids excessive manipulation of the tissues, providing durable and long-lasting results and reducing the likelihood of peripheral visual field compromise in the patient’s only functioning eye [4,9]. The curvilinear design allows adequate horizontal shortening and tarsal realignment while respecting the skin tension lines, thereby minimizing the risk of conspicuous scarring, dog-ear deformity, or cicatricial ectropion. The successful anatomical reconstruction, complete resolution of symptoms, and absence of complications in this case are consistent with the structural reliability of full-thickness wedge techniques [17], while illustrating the cosmetic and functional benefits of the lazy modification [10].

Finally, this case report broadens the potential indications for LPWR and suggests that it may represent a practical option for patients with severe involutional ectropion, significant horizontal laxity, generalized facial laxity, inflammation, or compromised periocular tissue integrity, particularly when preservation of peripheral vision and optimization of cosmetic outcome are paramount. The LPWR demonstrated excellent functional and cosmetic outcomes. In addition, the technique offered a durable and visually favorable result while avoiding the limitations of more traditional procedures.

## Conclusions

This novel application of the lazy pentagonal wedge resection method as a stand-alone procedure for the correction of severe involutional ectropion with marked horizontal laxity, generalized facial laxity, inflammation, and compromised periocular tissue integrity demonstrated excellent functional and cosmetic outcomes. By providing effective eyelid tightening, precise tarsal realignment, and improved incision results along relaxed skin tension lines, the technique offered an anatomically durable and visually favorable result while avoiding the limitations of more traditional procedures.
